# Epigenetic Aging Acceleration in Obesity Is Slowed Down by Nutritional Ketosis Following Very Low-Calorie Ketogenic Diet (VLCKD): A New Perspective to Reverse Biological Age

**DOI:** 10.3390/nu17061060

**Published:** 2025-03-18

**Authors:** Andrea G. Izquierdo, Paula M. Lorenzo, Nicolás Costa-Fraga, David Primo-Martin, Gemma Rodriguez-Carnero, Carolina F. Nicoletti, J. Alfredo Martínez, Felipe F. Casanueva, Daniel de Luis, Angel Diaz-Lagares, Ana B. Crujeiras

**Affiliations:** 1 Epigenomics in Endocrinology and Nutrition Group, Epigenomics Unit, Instituto de Investigacion Sanitaria de Santiago de Compostela (IDIS), Complejo Hospitalario Universitario de Santiago de Compostela (CHUS/SERGAS), 15706 Santiago de Compostela, Spain; andrea.gonzalez.izquierdo@hotmail.com (A.G.I.); paula.marino.lorenzo@sergas.es (P.M.L.); maria.gemma.rodriguez.carnero@sergas.es (G.R.-C.); felipe.casanueva@usc.es (F.F.C.); 2 CIBER Fisiopatologia de La Obesidad y Nutricion (CIBERobn), 28029 Madrid, Spain; 3 Cancer Epigenomics, Epigenomics Unit, Translational Medical Oncology Group (ONCOMET), Instituto de Investigacion Sanitaria de Santiago de Compostela (IDIS), Complejo Hospitalario Universitario de Santiago de Compostela (CHUS/SERGAS), Universidad de Santiago de Compostela (USC), 15706 Santiago de Compostela, Spain; nicocostafraga@gmail.com (N.C.-F.); adiazla@gmail.com (A.D.-L.); 4 CIBER de Cancer (CIBERonc), 28029 Madrid, Spain; 5 Center of Investigation of Endocrinology and Nutrition, Department of Endocrinology and Investigation, Medicine School, Hospital Clinico Universitario, University of Valladolid, 47011 Valladolid, Spain; dprimoma@saludcastillayleon.es (D.P.-M.); dluisro@saludcastillayleon.es (D.d.L.); 6 Applied Physiology and Nutrition Research Group, School of Physical Education and Sport, Faculdade de Medicina FMUSP, Universidade de Sao Paulo, Sao Paulo 05508-900, Brazil; carolnicolettifino@gmail.com; 7 Precision Nutrition Program, Research Institute on Food and Health Sciences IMDEA Food, CSIC-UAM, 28049 Madríd, Spain; jalfredo.martinez@imdea.org (J.A.M.); 8 Centre of Medicine and Endocrinology, University of Valladolid, 47002 Valladolid, Spain

**Keywords:** DNA methylation, body weight loss, personalized therapy, blood leukocytes, ketone bodies, epigenetic clock, Horvath, Hannum, Levine, precision medicine

## Abstract

**Background/Objectives**: Epigenetic clocks have emerged as a tool to quantify biological age, providing a more accurate estimate of an individual’s health status than chronological age, helping to identify risk factors for accelerated aging and evaluating the reversibility of therapeutic strategies. This study aimed to evaluate the potential association between epigenetic acceleration of biological age and obesity, as well as to determine whether nutritional interventions for body weight loss could slow down this acceleration. **Methods**: Biological age was estimated using three epigenetic clocks (Horvath (Hv), Hannum (Hn), and Levine (Lv)) based on the leukocyte methylome analysis of individuals with normal weight (n = 20), obesity (n = 24), and patients with obesity following a VLCKD (n = 10). We analyzed differences in biological age estimates, the relationship between age acceleration and obesity, and the impact of VLCKD. Correlations were assessed between age acceleration, BMI, and various metabolic parameters. **Results**: Analysis of the epigenetic clocks revealed an acceleration of biological age in individuals with obesity (Hv = +3.4(2.5), Hn = +5.7(3.2), Lv = +3.9(2.7)) compared to a slight deceleration in individuals with normal weight. This epigenetic acceleration correlated with BMI (*p* < 0.0001). Interestingly, patients with obesity following a VLCKD showed a deceleration in estimated biological age, both in nutritional ketosis (Hv = −3.3(4.0), Hn = −6.3(5.3), Lv = −8.8(4.5)) and at endpoint (Hv = −1.1(4.3), Hn = −7.4(5.6), Lv = −8.2(5.3)). Relevantly, this slowdown in age is associated with BMI (*p* < 0.0001), ketonemia (*p* ≤ 0.001), and metabolic parameters (*p* < 0.05). **Conclusions**: Our findings highlight the applicability of epigenetic clocks to monitor obesity-related biological aging in precision medicine and show the potential efficacy of the VLCKD in slowing obesity-related epigenetic aging.

## 1. Introduction

Obesity remains a significant and alarming global public health concern, not only due to obesity itself but also because it is the primary risk factor for an increasing array of chronic diseases and severe age-related conditions [1,2]. Concurrently, population aging represents a significant challenge for healthcare systems [3], which has driven growing interest in biomedical research regarding the relationship between obesity and aging in recent years. In this regard, it is known that obesity shares several biological traits with aging, primarily related to excessive accumulation of adipose tissue. These include the presence of low-grade chronic inflammation, oxidative stress, and mitochondrial dysfunction, all of which are considered hallmarks of aging [4,5]. These pathological conditions associated with obesity contribute to triggering aberrant epigenetic modifications, which also significantly impact the epigenetic patterns of aging, potentially accelerating this process at the cellular and molecular level [4,6,7]. However, the mechanistic link between aging and obesity is complex [8], so understanding the early etiology of the association between obesity and accelerated biological aging is crucial for mitigating the risk of future age-related diseases. Thus, in the last decade, various approaches have been considered to quantify biological aging and thereby evaluate its link with obesity, achieving significant advances in this line mainly due to the analysis of the epigenetic clock, developed to shed light on the aging process, currently being the most promising predictor of biological age [9].

Epigenetic clocks have emerged as a potential molecular tool for measuring biological aging, offering a deeper perspective on this process and its implications for health [10,11,12]. These instruments, designed to predict biological age based on DNA methylation patterns, have evolved from the first-generation clocks of Horvath and Hannum [13,14] to the second-generation clock of Levine, defined as PhenoAge [15]. These clocks used as reference estimators in most recent research, allow for determining epigenetic age acceleration, defined as the discrepancy between measured biological age and chronological age, to examine possible associations between accelerated biological aging and chronic pathologies such as obesity [6,16,17,18]. In fact, recent studies have shown that obesity is associated with accelerated aging, significantly linked to increases in BMI, and is especially detected in metabolically active tissues such as adipose tissue, liver, or blood [19,20,21,22,23]. This field of research is constantly evolving, so advancing the understanding of molecular mechanisms linking obesity with accelerated aging opens new avenues for developing therapies aimed at reversing or slowing this process. These advances exhibit a promising outlook for the future of personalized anti-aging medicine, where the analysis of epigenetic clocks, postulated as potential new biomarkers, offers a valuable tool for precisely measuring biological age and evaluating the impact of various interventions on the aging process, such as lifestyle changes based on diet and exercise [24,25,26,27].

In this context of developing more personalized and effective therapies to address biological aging and its health consequences, we wonder whether body weight loss interventions, such as the very-low-calorie ketogenic diet (VLCKD), could be potential strategies to contribute to the deceleration of obesity-associated epigenetic aging. There is a lot of evidence supporting the effectiveness and safety of this therapeutic strategy for the treatment of obesity and its associated comorbidities since, in addition to achieving significant weight loss, it also improves cardiometabolic profiles [28,29,30,31,32,33], which is why European guidelines recommend its applicability in the management of obesity in adults [34]. Furthermore, in previous work, we have determined the beneficial effects of VLCKD regarding improvements in body composition, metabolic health and quality of life [35], and more specifically, in relation to this issue, we have recently demonstrated the reversal of methylome and the immunomodulatory effect on markers of oxidative and inflammatory stress in patients with obesity who followed this diet [36,37]. Therefore, based on our findings, the main objective of the present work has been to investigate whether the acceleration of biological age induced by obesity could be reversed by following a nutritional therapy for body weight loss based on a VLCKD in patients with obesity. For this purpose, biological age was quantified through the analysis of epigenetic age estimated by Horvath, Hannum, and Levine clocks, and epigenetic acceleration was analyzed based on the difference with chronological age. Additionally, the possible association of epigenetic age acceleration with BMI and other metabolic parameters was determined. To carry out this study, a cross-sectional cohort of people with obesity compared to people with normal weight and a longitudinal cohort of patients with obesity who underwent a VLCKD were recruited.

## 2. Materials and Methods

### 2.1. Study Populations

Cross-sectional cohort participants were classified according to their body mass index (BMI) into two groups: obesity group (n = 28; age = 36.4(8.15) years), including individuals with a BMI > 30 kg/m^2^, and normal-weight group (n = 20; age = 38.7(13.5) years), including individuals with a BMI between 20–25 kg/m^2^ (Table S1).

Longitudinal cohort participants included patients with obesity (n = 10; age = 49.3 (8.9) years) following a nutritional intervention for body weight loss based on a very low-calorie ketogenic diet (VLCKD) for 6 months (180 days) (Table S2). The nutritional intervention was based on a commercial body weight loss program consisting of five steps (PNK^®^ method) [36]. Epigenetic analyses were performed using blood samples collected at different times throughout the diet follow-up, specifically at day 0 (Baseline, BL), at 30 days (Nutritional Ketosis, NK), and at 180 days (Endpoint, EP) from the start of body weight loss therapy.

All participants were European Caucasians who underwent a comprehensive evaluation, which included medical history, physical examination, and standard laboratory tests. All anthropometric measurements were performed in duplicate and at rest, and blood samples for genomic DNA extraction were collected in specific tubes (EDTA Vacutainer Tubes, BD Biosciences, Franklin Lakes, NJ, USA), all under conditions of overnight fasting (8 to 10 h). Body mass index (BMI) was calculated as body weight in kilograms divided by the square of height in meters (BMI = weight (kg)/height (m^2^)).

Participants reported no significant weight fluctuations or use of vitamin and/or mineral supplements or prescribed medications during the previous three months. Individuals with pregnancy, substance abuse, or chronic medication use (except for antidiabetic therapy) were excluded.

The research protocols from which data were collected were approved by the corresponding Clinical Research Ethics Committees in each study (Ethical Committee of Clinical Research of Galicia; Ethical Committee of the University Hospital of Valladolid) and adhered to the recommendations of the Declaration of Helsinki. All participants provided written informed consent.

### 2.2. Blood DNA Methylation Dataset and Epigenetic Age

Genomic DNA from peripheral blood circulating leukocyte samples was purified using the MasterPure DNA Extraction Kit (Epicentre Biotechnologies, Madison, WI, USA) according to the manufacturer’s instructions. Bisulfite-converted DNA, processed with the EZ DNA Methylation Kit (Zymo Research, Irvine, CA, USA), was hybridized to Infinium HumanMethylation450 BeadChip arrays (cross-sectional cohort) and Infinium MethylationEPIC BeadChip arrays (longitudinal cohort), following the manufacturer’s Infinium HD Methylation protocol (Illumina, San Diego, CA, USA). Detection was performed on the HiScan SQ System platform (Illumina). DNA methylation status visualization, data processing, quality control, and normalization were evaluated using GenomeStudio software (V2011.1) (Illumina) and BeadArray Controls Reporter (Illumina) in accordance with previously described experimental procedures [38,39]. The methylation score for each valid cytosine-phosphate-guanine (CpG) site was quantitatively calculated from the ratio of fluorescent signals of methylated to unmethylated sites and expressed as a beta value (β), ranging from 0 (completely unmethylated) to 1 (completely methylated).

From the methylation status of each valid CpG, extracted from the methylome data generated in our previously published [36,40] and unpublished studies, we proceeded to estimate the DNA methylation-based epigenetic age (DNAmAge). This study selected three of the most widely used and validated epigenetic clocks, specifically, the first-generation clocks by Horvath [13] and Hannum [14] and the second-generation clock by Levine, also known as PhenoAge [15]. The Horvath and Hannum estimators predict biological age based on DNA methylation levels at 353 and 71 specific CpG positions, respectively. The Levine estimator predicts a measure of phenotypic age based on DNA methylation levels at 513 CpG sites. Epigenetic age measures for each estimator were generated using an online calculator (https://dnamage.genetics.ucla.edu/new, accessed on 3 March 2024) [13].

To determine whether study participants were biologically younger or older compared to their chronological age, age acceleration (AgeAccel) was calculated as the difference between an individual’s DNAmAge and their chronological age (ChronoAge) for each of the analyzed clocks. AgeAccel was used as an indicator of aging status, where a positive value was considered accelerated aging, while a negative value was considered slower aging [19,41].

### 2.3. Statistical Analysis

Valid CpG sites, according to quality control, were previously filtered using Genome Studio Illumina software (V2010.3). For each of the above-mentioned epigenetic clocks used as biological age estimators, simple linear regression analyses were performed to estimate the relationship between chronological age (ChronoAge) and DNAmAge in each study group and cohort.

The estimate of epigenetic AgeAccel was calculated as the difference between each individual’s DNAmAge and their ChronoAge in each of the participants included in the study cohorts. Differences in age acceleration for each estimator between the groups of the cross-sectional cohort were analyzed by the paired *t*-test corrected for multiple comparisons using the Holm-Šídák method. Differences in age acceleration were analyzed at the baseline, maximum nutritional ketosis, and endpoints in the VLCKD of the longitudinal cross-sectional cohort using a repeated measures ANOVA test and a Bonferroni post-hoc analysis.

The association between age acceleration (AgeAccel), body mass index (BMI), and metabolic parameters was assessed using the Spearman correlation coefficient for each epigenetic clock and in both study cohorts.

All data are expressed as mean ± standard deviation (SD) in the tables and as mean ± standard error of the mean (SEM) in the figures. *p* < 0.05 was considered statistically significant. Data analysis was carried out using R version 4.2.0 (R Foundation for Statistical Computing, Vienna, Austria), IBM SPSS Statistics version 25.0 (SPSS Inc., Chicago, IL, USA) and GraphPad Prism version 9.5.1 (GraphPad Software, San Diego, CA, USA).

## 3. Results

### 3.1. Epigenetic Aging Is Accelerated by Obesity and Correlates with BMI

#### 3.1.1. Individuals with Obesity Exhibit Higher Biological Age (DNAmAge) Compared to Healthy Normal-Weight Subjects

**Figure 1:**
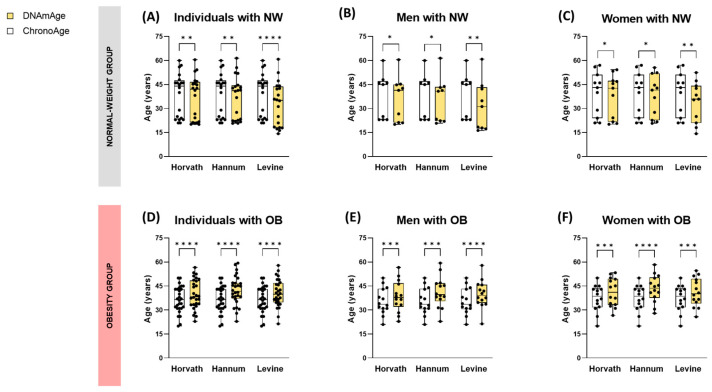
Analysis of the epigenetic clock in the cross-sectional cohort. Boxplots of chronological age (ChronoAge) (white) versus DNA methylation age (DNAmAge) (yellow) values, estimated by the 3 epigenetic predictors, comparing individuals with normal-weight (**A**–**C**) versus individuals with obesity (**D**–**F**). Each bar graph represents the mean value, standard deviation, and asterisks indicating significant differences according to paired *t*-test corrected for multiple comparisons using the Holm-Šídák method (*: *p* < 0.05; **: *p* < 0.01; ***: *p* < 0.001; ****: *p* < 0.0001).

Epigenetic age was assessed using blood DNA methylation data, employing three distinct methodologies: the Horvath, Hannum, and Levine clocks. The initial analysis of the cross-sectional cohort (Table S1) revealed significant differences between chronological age (ChronoAge) and estimated biological age (DNAmAge) across all three clocks. Notably, the obesity group demonstrated a higher DNAmAge, while the normal-weight group showed a lower DNAmAge. The magnitude of these differences was more pronounced in the obesity group for all clocks (*p* < 0.0001) (Table S3) (Figure 1A,D).

When evaluating the differences observed by stratifying individuals according to sex, the discrepancies detected in the groups between DNAmAge and ChronoAge were similar when comparing men and women (*p* < 0.05 in the normal-weight group; *p* < 0.001 in the obesity group) (Figure 1B,C,E,F).

#### 3.1.2. Epigenetic Age Acceleration (AgeAccel) in Obesity Is Associated with BMI

**Figure 2:**
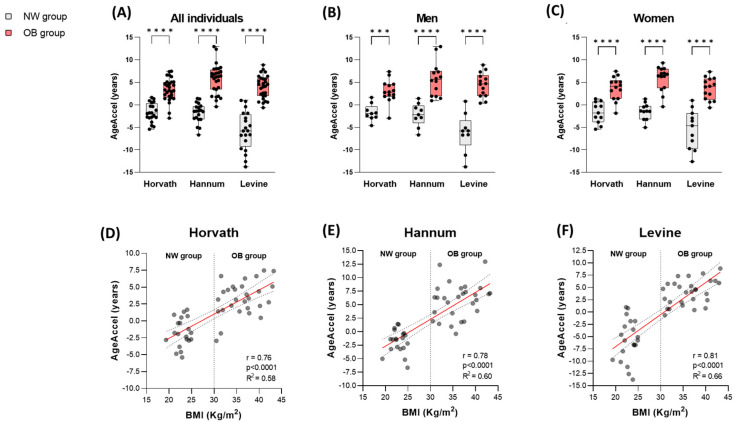
Analysis of the acceleration of biological age in the cross-sectional cohort. Boxplots of epigenetic age acceleration (AgeAccel) values in normal-weight individuals (gray) versus individuals with obesity (red), estimated by the 3 epigenetic predictors (**A**–**C**). Each bar graph represents the mean value standard deviation, and asterisks indicate significant differences according to the paired *t*-test corrected for multiple comparisons using the Holm-Šídák method (***: *p* < 0.001; ****: *p* < 0.0001). Scatter plot with a line of best fit showing the association of age acceleration (AgeAccel) with body mass index (BMI) using the Spearman correlation coefficient (r) (*p*-value < 0.05) for each of the epigenetic clocks (**D**–**F**).

The observed differences between ChronoAge and DNAmAge manifested as an acceleration of biological age (AgeAccel) in individuals with obesity, contrasting with a slight deceleration detected in normal-weight subjects (Table S3). Specifically, the AgeAccel in the obesity group was significantly higher compared to the normal-weight group across all three estimators (*p* < 0.0001). On average, individuals with obesity exhibited an AgeAccel of +4.4 years, while normal-weight individuals showed a deceleration of −3.1 years (Table S3). Furthermore, this statistical significance persisted when subjects were analyzed separately by sex (*p* < 0.001 in men; *p* < 0.0001 in women) (Figure 2A–C).

Correlation analyses were conducted between AgeAccel and BMI to explore the relationship between age acceleration and body composition. The results revealed a statistically significant positive correlation for all three estimators (r(Hv) = 0.76, *p* < 0.0001; r(Hn) = 0.78, *p* < 0.0001; r(Lv) = 0.81, *p* < 0.0001). This strong positive association between increased BMI and AgeAccel suggests that obesity is linked to accelerated biological aging (Figure 2D–F).

These findings underscore the impact of obesity on epigenetic aging processes and highlight the potential use of epigenetic clocks as biomarkers for obesity-related health risks.

### 3.2. VLCKD Slows Down Obesity-Associated Epigenetic Aging Through Nutritional Ketosis

#### 3.2.1. DNAmAge Is Reduced in Patients with Obesity Following a Nutritional Body Weight Loss Therapy

A reduction in DNAmAge was observed in patients with obesity following a nutritional intervention based on a VLCKD. The DNAmAge was estimated using the Horvath, Hannum, and Levine epigenetic clocks, derived from methylome data of blood samples collected from subjects in a longitudinal cohort (Table S2).

**Figure 3:**
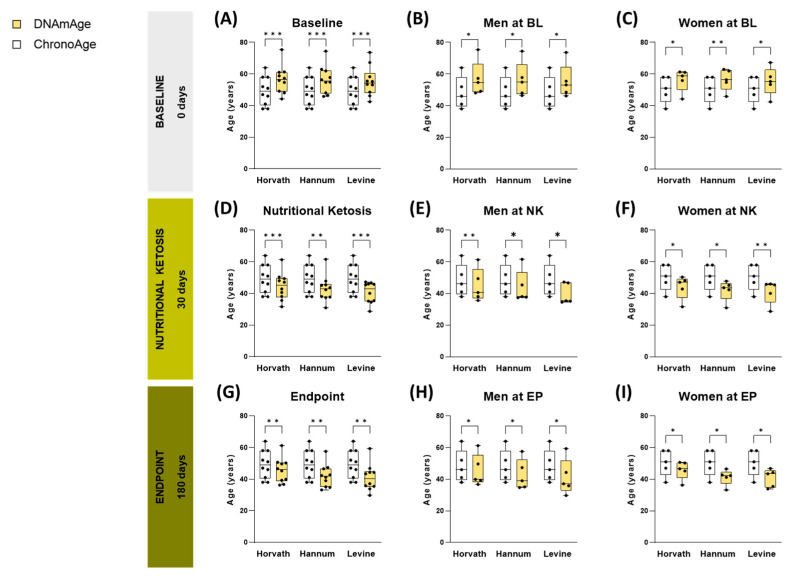
Analysis of the epigenetic clock in the longitudinal cohort. Boxplots of chronological age (ChronoAge) (white) versus DNA methylation age (DNAmAge) (yellow) values, estimated by the 3 epigenetic predictors, comparing the different phases of the follow-up of a VLCKD in patients with obesity: baseline (BL) (**A**–**C**), nutritional ketosis (NK) (**D**–**F**) and endpoint (EP) (**G**–**I**). Each bar graph represents the mean value standard deviation, and asterisks indicate significant differences according to the paired *t*-test corrected for multiple comparisons using the Holm-Šídák (*: *p* < 0.05; **: *p* < 0.01; ***: *p* < 0.001).

The analysis revealed significant changes in DNAmAge of patients with obesity after the VLCKD intervention (Figure 3). Particularly, DNAmAge showed a marked decrease compared to ChronoAge across all three epigenetic clocks (*p* < 0.005 for all comparisons) (Table S4). This reduction was evident both during the nutritional ketosis phase (30 days into the diet, with an average body weight loss of 9 kg) and at the end of the intervention (180 days, with an average body weight loss of 20 kg) (Table S4) (Figure 3A,D,G).

When stratified by sex, the decrease in DNAmAge relative to ChronoAge was consistent between men and women across all time points analyzed during the diet (Figure 3B,C,E,F,H,I). Additionally, it is noteworthy that at baseline, the DNAmAge of patients with obesity was higher than their ChronoAge, mirroring findings from the cross-sectional cohort of individuals with obesity (Figure 1D–F and Figure 3A–C).

#### 3.2.2. DNAmAge Deceleration Is Associated with Nutritional Ketosis and Improvement in Metabolic Health in Patients with Obesity During Diet

**Figure 4:**
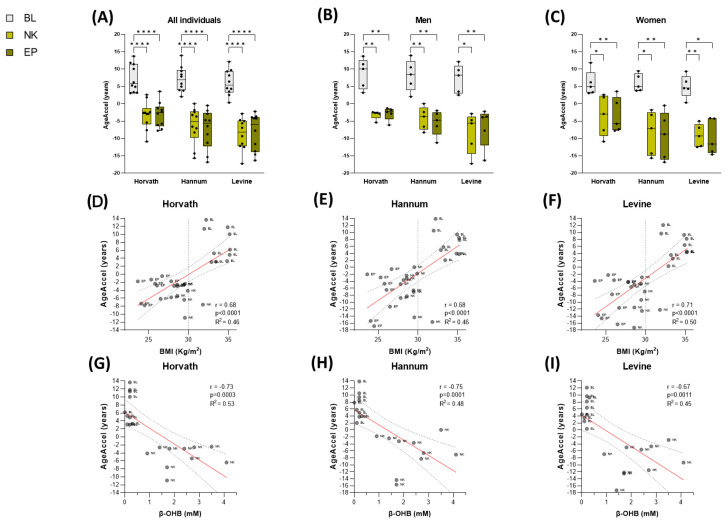
Analysis of the acceleration of biological age in the longitudinal cohort. Boxplots of epigenetic age acceleration (AgeAcell) values in patients with obesity following a VLCKD, comparing baseline (BL) (gray), nutritional ketosis (NK) (light green) and endpoint (EP) (dark green), estimated by the 3 epigenetic predictors, using a repeated measures ANOVA test applying a Bonferroni post hoc analysis (*: *p* < 0.05; **: *p* < 0.01; ****: *p* < 0.0001) (**A**–**C**). Scatter plot with a line of best fit showing the association of age acceleration (AgeAccel) with body mass index (BMI) (**D**–**F**) and with β-hydroxybutyrate (β-OHB) levels (**G**–**I**), using the Spearman correlation coefficient (r) (*p*-value < 0.05) for each of the epigenetic clocks.

Notably, the VLCKD evidenced a significant deceleration in DNAmAge. Specifically, during the nutritional ketosis phase, which occurred approximately 30 days into the VLCKD intervention, a notable slowdown in average epigenetic age was observed, an average age slowdown of −6.1 years (*p* < 0.0001). This deceleration persisted until the final phase of the intervention (180 days), with an average value of −6.2 years (*p* < 0.0001) (Table S4) (Figure 4A). The age deceleration followed a similar pattern when comparing estimates between men and women (Figure 4B,C). These results indicate a partial reversal of the accelerated aging initially observed in patients with obesity. This reversal significantly correlated with changes in subjects’ BMI linked to body weight loss achieved after the diet (r(Hv) = 0.68, *p* < 0.0001; r(Hn) = 0.68, *p* < 0.0001; r(Lv) = 0.71, *p* < 0.0001) (Figure 4D–F).

To determine whether this slowdown in aging could be linked to nutritional ketosis, a characteristic phase of this type of therapy, the potential association with ketone bodies in capillary blood was analyzed. Specifically, β-hydroxybutyrate (β-OHB) levels were measured at 30 days of diet adherence. Interestingly, the results showed a statistically significant negative correlation between higher ketonemia and lower AgeAccel estimated by the three epigenetic clocks used in the study (r(Hv) = −0.73, *p* = 0.0003; r(Hn) = −0.75, *p* = 0.0001; r(Lv) = −0.67, *p* = 0.0011) (Figure 4G–I). This finding suggests that the presence of ketone bodies, particularly β-OHB, during the nutritional ketosis phase may be associated with the observed deceleration in epigenetic aging.

Furthermore, given our previous findings that adherence to a VLCKD positively impacts the metabolic health of patients, we sought to investigate whether the observed attenuation in AgeAccel could be associated with improvements in biochemical parameters resulting from the dietary intervention. To elucidate this potential relationship, we analyzed the association between AgeAccel and blood levels of routine biochemical parameters. Specifically, we examined glucose, insulin, AST (Aspartate Aminotransferase), ALT (Alanine Aminotransferase), GGT (Gamma-Glutamyl Transferase), total cholesterol, triglycerides, LDL (Low-Density Lipoprotein) cholesterol, HDL (High-Density Lipoprotein) cholesterol, leptin, resistin, adiponectin, waist circumference and percentage of body weight loss. These parameters were measured in patients with obesity at different time points throughout the dietary intervention: at baseline, during the ketosis phase, and at the conclusion of the diet.

Our analysis revealed that the reduction in AgeAccel was indeed associated with improvements in the biochemical profiles of patients with obesity following the VLCKD intervention. Notably, we observed statistically significant positive correlations between the deceleration estimated by all three epigenetic clocks and several key metabolic markers, including glucose (r(Hv) = 0.55, *p* = 0.0015; r(Hn) = 0.51, *p* = 0.0038; r(Lv) = 0.61, *p* = 0.0003), insulin (r(Hv) = 0.66, *p* < 0.0001; r(Hn) = 0.63, *p* = 0.0002; r(Lv) = 0.78, *p* < 0.0001), cholesterol (r(Hv) = 0.53, *p* = 0.0031; r(Hn) = 0.48, *p* = 0.0087; r(Lv) = 0.40, *p* = 0.0310), triglycerides (r(Hv) = 0.50, *p* = 0.0045; r(Hn) = 0.49, *p* = 0.0058; r(Lv) = 0.38, *p* = 0.0384), leptin (r(Hv) = 0.46, *p* = 0.0114; r(Hn) = 0.44, *p* = 0.0139; r(Lv) = 0.52, *p* = 0.0035), waist circumference (r(Hv) = 0.40, *p* = 0.0319; r(Hn) = 0.51, *p* = 0.0038; r(Lv) = 0.61, *p* = 0.0003) and % body weight loss (r(Hv) = 0.59, *p* = 0.0007; r(Hn) = 0.66, *p* < 0.0001; r(Lv) = 0.62, *p* = 0.0003) (Table 1).

To further discard the effect of nutritional ketosis from the effect of body weight loss, we compared the deceleration in DNAmAge achieved after VLCKD with other body weight loss interventions such as bariatric surgery. Thus, based on the methylome data obtained in a previous study carried out in women with obesity undergoing this surgical intervention [42], we analyzed AgeAccel based on the biological age estimated by the three epigenetic clocks used. Interestingly, we did not observe significant changes regarding a slowdown in AgeAcell after surgery (*p*(Hv)= 0.3659; *p*(Hn) = 0.5664; *p*(Lv) = 0.9697), data that agreed with another study on aging in severe obesity after bariatric surgery [43].

## 4. Discussion

The present study demonstrated the efficacy of a very low-calorie ketogenic diet (VLCKD) in slowing the biological age of patients with obesity undergoing this nutritional therapy for body weight loss. Our results revealed that the benefits induced by a VLCKD in obesity treatment were associated with a deceleration of obesity-related epigenetic aging following adherence to the diet. Interestingly, a significant correlation was observed between increased levels of ketone bodies and the reduction in epigenetic age acceleration (AgeAccel), potentially linked to the effect of nutritional ketosis, characteristic of this intervention, alongside the significant body weight loss achieved. Furthermore, this deceleration was also associated with reductions in BMI and improvements in blood levels of metabolic health parameters, such as glucose, insulin, cholesterol, and triglycerides. Notably, this is the first study to highlight a possible relationship between adherence to a VLCKD and the reversal of biological aging in patients with obesity. Taken together, our study underscores the potential of nutritional strategies like VLCKD to counteract the impact of obesity on epigenetic aging.

Our study highlights the detrimental impact of obesity on biological aging and emphasizes the applicability of epigenetic clocks as potential biomarkers for monitoring aging acceleration associated with risk factors such as obesity [24]. Three well-established epigenetic clocks, specifically Horvath, Hannum, and Levine, were employed to evaluate DNA methylation age (DNAmAge) [13,14,15]. These DNA methylation-based estimators of epigenetic aging have demonstrated strong predictive capacities in previous studies on chronic diseases and mortality [44,45]. In the obesity context, research conducted in recent years has described accelerated epigenetic aging in individuals with obesity, which has been positively associated with BMI [19,23,46,47]. In fact, previous studies have shown that obesity exacerbates aging-associated epigenetic changes, resulting in an apparent acceleration equivalent to 2.7 years for every 10-point increase in BMI [20].

Consistent with these findings, our cross-sectional cohort analysis revealed an increased DNAmAge in individuals with obesity relative to their chronological age (ChronoAge), which was significantly higher when compared to the DNAmAge estimated in normal-weight subjects, who exhibited a slight decrease relative to their ChronoAge. Interestingly, individuals with obesity showed an AgeAccel > 4 years on average, as estimated by the three epigenetic clocks used. Furthermore, it is important to highlight that the detected AgeAccel exhibited a strong positive correlation with the individuals’ BMI, with significant correlation coefficients across all three estimators used. This correlation was stronger than those previously reported in the literature, such as r < 0.10 in blood [23] or r = 0.42 in liver [20]. These findings underscore the robustness of the relationship between BMI and accelerated epigenetic aging in our cohort and are in line with previous evidence that obesity is associated with accelerated biological aging [20,21,22,46,47,48,49], highlighting the potential use of epigenetic clocks as biomarkers of health risks related to obesity.

To determine if the metabolic benefits induced by VLCKD could contribute to reversing accelerated epigenetic aging detected in individuals with obesity, we explored the dynamics of biological aging in patients with obesity during this nutritional therapy. Notably, we detected a significant reduction in DNAmAge in our longitudinal cohort after the diet, revealing a remarkable deceleration in AgeAccel (>6 years). This deceleration of biological age was evident both during the nutritional ketosis phase (30 days of diet, with an average body weight loss of 9 kg) and at the end of the intervention (180 days, with an average body weight loss of 20 kg), indicating that the observed slowdown in ketosis was maintained after the progressive reintroduction of food and the increase in the percentage of carbohydrates and caloric intake, corresponding to the final phases of the dietary program [36]. Furthermore, this slowdown in biological age was significantly correlated with the reduction in BMI and several metabolic parameters achieved by the diet.

To contextualize these results, we suggest that the observed deceleration of epigenetic aging could be attributed to the significant benefits that VLCKD provides in various aspects of health and general well-being. Previous studies have demonstrated that this dietary intervention produces notable improvements in body composition, mainly in the reduction of visceral fat, which is metabolically more active and harmful, while preserving muscle mass, which is crucial for healthy aging [35]. Additionally, improvements were observed in other health indicators such as sleep quality, sexual function, and overall quality of life, aspects that are typically negatively affected with advancing age but show notable improvement with VLCKD [35]. Furthermore, our data reveal a possible mechanistic link, epigenetically regulated, connecting the metabolic improvements induced by VLCKD with the observed deceleration in biological aging. This approach is based on the detection of significant changes in DNA methylation patterns after VLCKD intervention, specifically located in 786 genes involved in crucial biological processes such as adipose tissue function, neurological functioning, muscle development, and other relevant metabolic processes [36]. These new data provide additional information to understand how this nutritional therapy can influence complex biological processes related to aging.

A particularly interesting finding was that the deceleration we detected with the diet was also significantly associated with ketonemia, suggesting that the production of ketone bodies in the form of β-hydroxybutyrate during the nutritional ketosis phase could be responsible. This relevant finding aligns with previous evidence where we demonstrated that nutritional ketosis was associated with benefits beyond body weight loss, including improvements in cognitive performance, reduction of inflammation and oxidative stress, and enhanced immunomodulatory capacity, among others [37]. In this sense, ketone bodies, such as beta-hydroxybutyrate, have recently also been related to beneficial health effects such as antioxidant capacity [50,51], improvement of insulin sensitivity [52], neuroprotection [53], improvement of liver function [54,55] or promotion of autophagy [56]. Furthermore, recent studies have also suggested that endogenous production of ketone bodies promotes longevity since it increases lifespan and survival [57,58,59].

On the other hand, the intestinal microbiota plays an important role in the regulation of metabolism and aging [60,61], indicating that the effects of VLCKD on epigenetic aging could also be related to the changes in the microbiome after the diet. Regarding this, several studies have shown that VLCKD can modulate the composition of the intestinal microbiome, promoting an improved anti-inflammatory and metabolic profile [62,63,64,65]. This evidence regarding the improvement of parameters related to healthy aging due to the change in the intestinal microbiota after VLCKD is an interesting aspect to consider in future research aimed at evaluating the role of the microbiome in epigenetic aging in patients undergoing a VLCKD.

**Figure 5:**
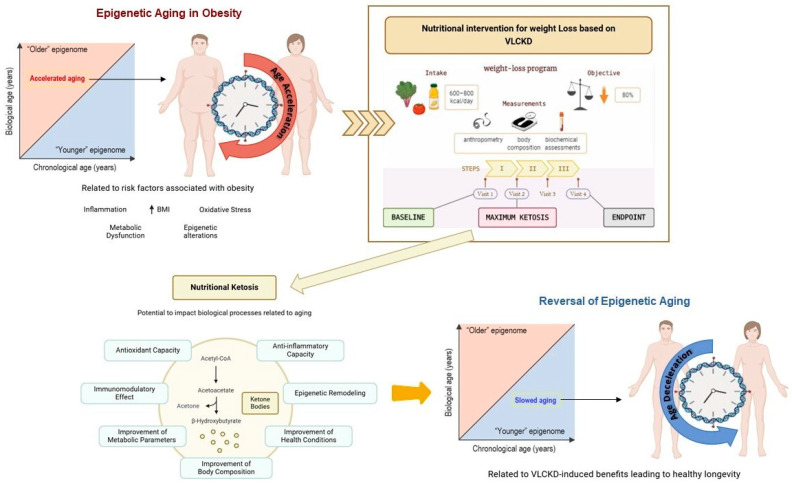
Summary. Schematic representation illustrating, according to our findings, the possible mechanistic link between metabolic improvements derived from nutritional ketosis induced by VLCKD and the slowing of obesity-related epigenetic aging.

Notably, this significant deceleration of estimated biological age in patients with obesity undergoing a VLCKD has not been observed in other body weight loss interventions. Specifically, after bariatric surgery, despite achieving significant and comparable body weight loss to that achieved with VLCKD, no notable differences were detected in the decrease in biological age of patients with obesity after surgical intervention [43]. These results are also in line with previous studies where we determined that VLCKD was able to modulate inflammatory state oxidative stress and improve innate immunity more effectively than with a hypocaloric diet or bariatric surgery [37]. Therefore, based on these observations, we propose that the effect of reversing the acceleration of biological age in patients with obesity undergoing VLCKD could be primarily mediated by diet-induced ketosis through epigenetic reprogramming or resetting. Overall, our findings underscore the therapeutic potential of VLCKD in the treatment of obesity, beyond body weight loss, as this nutritional strategy not only provides metabolic, anti-inflammatory, and antioxidant benefits but also and relevantly may have the capacity to modulate biological aging through epigenetic mechanisms, postulating it as a comprehensive therapeutic tool in the management of obesity and its associated complications (Figure 5).

Our study has several strengths, including the use of multiple epigenetic clocks, a longitudinal design, and the assessment of various metabolic parameters. However, certain limitations should be considered. The study population consisted primarily of middle-aged adults with obesity, which may somewhat limit the generalizability of our findings. Furthermore, while we observed significant correlations, definitive causal relationships cannot be established without additional mechanistic studies. Therefore, our findings regarding the strong association between nutritional ketosis and the deceleration of obesity-linked epigenetic age are suggested as a potential mechanism of VLCKD beyond body weight loss. Accordingly, we consider future research, including longitudinal studies with larger and more diverse populations at other centers and with long-term patient follow-up, as well as the possibility of elucidating deeper molecular mechanisms underlying our findings on epigenetic aging slowing from preclinical models of animal experiments supplemented with ketone body.

## 5. Conclusions

In conclusion, this study reinforces the importance of utilizing epigenetic clocks for monitoring obesity-associated epigenetic aging, with the aim of advancing toward personalized and precision medicine. Our findings highlight the potential applicability of these estimators as potential epigenetic biomarkers, as they are postulated to be valuable future tools for evaluating the effects of dietary interventions on biological aging in individuals with obesity. Relevantly, this study demonstrates that adherence to a VLCKD in patients with obesity is associated with a deceleration of aging, opening a promising avenue for exploring the beneficial effects of nutritional ketosis on extending the life expectancy of these patients. Our findings provide novel and valuable information in this field, supporting the need to carry out new studies with larger sample sizes and longer follow-up times that will reinforce and consolidate the role of ketone bodies in the epigenetic regulation of aging. Finally, it is worth emphasizing that, to date, this is the first study to provide evidence for the potential of a VLCKD to delay the aging process, suggesting a sensitive effect of nutritional ketosis on recognized obesity-related biological aging processes.

## Figures and Tables

**Table 1:** Association of age acceleration (AgeAccel) with metabolic parameters in the longitudinal cohort of patients with obesity following a very low-calorie ketogenic diet (VLCKD), estimated by epigenetic predictors.

		Glu	Ins	AST	ALT	GGT	TC	HDL	LDL	TG	Lep	Res	Adi	WC	%BWL
AgeAccel Horvath	r	0.555	0.663	0.017	0.273	0.551	0.530	0.182	0.215	0.504	0.456	−0.295	−0.193	0.393	0.585
	*p*	0.0015	<0.0001	0.9303	0.1451	0.0016	0.0031	0.3442	0.2625	0.0045	0.0114	0.1132	0.306	0.0319	0.0007
	sig.	**	****	ns	ns	**	**	ns	ns	**	*	ns	ns	*	***
AgeAccel Hannum	r	0.513	0.630	−0.029	0.314	0.510	0.478	0.085	0.156	0.492	0.444	−0.270	−0.170	0.572	0.664
	*p*	0.0038	0.0002	0.8785	0.0907	0.004	0.0087	0.6618	0.4199	0.0058	0.0139	0.1486	0.3686	0.001	<0.0001
	sig.	**	***	ns	ns	**	**	ns	ns	**	*	ns	ns	***	****
AgeAccel Levine	r	0.614	0.777	−0.097	0.206	0.486	0.401	0.076	0.082	0.380	0.516	−0.138	−0.268	0.449	0.621
	*p*	0.0003	<0.0001	0.6109	0.2742	0.0065	0.031	0.6954	0.6713	0.0384	0.0035	0.4666	0.1528	0.0127	0.0003
	sig.	***	****	ns	ns	**	*	ns	ns	*	**	ns	ns	*	***

AgeAccel: age acceleration; Glu: Glucose; Ins: Insulin; AST: Aspartate Aminotransferase; ALT: Alanine Aminotransferase; GGT: Gamma-Glutamyl Transferase; TC: Total Cholesterol; HDL: High-Density Lipoprotein Cholesterol; LDL: Low-Density Lipoprotein Cholesterol; TG: Triglycerides; Lep: Leptin; Res: Resistin; Adi: Adiponectin; WC: Waist Circumference; %BWL: Percentage of Body Weight Loss; r: Spearman correlation coefficient; *p*: *p*-value; sig.: significant; ns: not significant; *: *p* < 0.05; **: *p* < 0.01; ***: *p* < 0.001; ****: *p* < 0.0001.

## Data Availability

The data presented in this study are available at the request of the corresponding authors due to privacy and ethical restrictions.

## Supplementary Materials

The following supporting information can be downloaded at: https://www.mdpi.com/article/10.3390/nu17061060/s1, Table S1: Anthropometric Parameters of Subjects Included in the Cross-Sectional Cohort.; Table S2: Anthropometric Parameters of Subjects Included in the Longitudinal Cohort; Table S3: Data from the comparison between ChronoAge and DNAmAge and the calculation of AgeAccel, estimated by each epigenetic predictor, of the subjects included in the cross-sectional cohort; Table S4: Data from the comparison between ChronoAge and DNAmAge and the calculation of AgeAccel, estimated by each epigenetic predictor, of the subjects included in the longitudinal cohort.
