# Supplementary material for: Epigenetic Aging Acceleration in Obesity Is Slowed Down by Nutritional Ketosis Following Very Low-Calorie Ketogenic Diet (VLCKD): A New Perspective to Reverse Biological Age
Source: Nutrients. 2025 Mar 18;17(6):1060. doi: 10.3390/nu17061060 (PMC11945372; doi:10.3390/nu17061060)
Supplement: Supplementary file 1 [file nutrients-17-01060-s001.zip › nutrients-3519048-supplementary.pdf]

Table S1. Anthropometric Parameters of Subjects Included in the Cross-Sectional Cohort.

	Cross-sectional Cohort						
**Group**	**NORMAL-WEIGHT**			**OBESITY**			
**n**	20			28			
**Sex (Men/Women)**	9/11			14/14			
	**Mean**	**Median**	**SD**	**Mean**	**Median**	**SD**	**p-value**
**Age (years)**	38.7	44	13.5	36.4	36.5	8.15	0.4814
**BMI (kg/m^2)**	22.9	22.8	1.45	36.2	36.49	3.91	<0.0001

Abbreviations: BMI, Body Mass Index; SD, Standard Deviation.

Table S2. Anthropometric Parameters of Subjects Included in the Longitudinal Cohort.

	**Longitudinal Cohort**										
**n**	10										
**Sex (Men/Women)**	5/5										
**Phase of VLCKD**	**BL**			**NK**			**EP**			**BL-NK**	**BL-EP**
	**Mean**	**Median**	**SD**	**Mean**	**Median**	**SD**	**Mean**	**Median**	**SD**	**p-value**	**p-value**
**Age (years)**	49.3	49.0	8.93	49.3	49.0	8.93	49.3	49.0	8.93	-	-
**BMI (kg/m^2)**	33.9	34.1	1.3	29.4	29.5	1.03	25.6	25.9	1.67	<0.0001	<0.0001
**%BWL**	-	-	-	-13.3	-8.13	3.18	-24.4	-5.99	4.81	<0.0001	<0.0001

Abbreviations: VLCKD; very low-calorie ketogenic diet; BMI, Body Mass Index; %BWL: Percentage of Body Weight Loss; BL, baseline; NK, nutritional ketosis; EP, endpoint; SD, Standard Deviation.

Table S3. Data from the comparison between ChronoAge and DNAmAge and the calculation of AgeAccel, estimated by each epigenetic predictor, of the subjects included in the cross-sectional cohort.

NORMAL-WEIGHT GROUP											
ChronoAge					DNAmAge						
Mean	Median	SD	Max	Min		Mean	Median	SD	Max	Min	Adj. p-value
**38.65**	44.00	13.50	60.00	21.00	**Horvath**	36.93	42.02	13.48	60.51	20.10	0.0031
					**Hannum**	36.86	41.07	13.20	61.43	20.73	0.0031
					**Levine**	32.87	35.11	13.73	60.78	14.24	<0.0001

OBESITY GROUP											
ChronoAge					DNAmAge						
Mean	Median	SD	Max	Min		Mean	Median	SD	Max	Min	Adj. p-value
**36.43**	36.50	8.15	50.00	20.00	**Horvath**	39.80	38.27	9.20	56.62	22.89	<0.0001
					**Hannum**	42.23	41.66	8.98	59.38	22.87	<0.0001
					**Levine**	40.44	39.87	8.60	57.87	21.39	<0.0001

AgeAccel							
	NORMAL-WEIGHT GROUP			OBESITY GROUP			Adj. p-value
	Mean	Median	SD	Mean	Median	SD	
**Horvath**	-1.72	-1.86	2.10	3.37	3.25	2.54	<0.0001
**Hannum**	-1.79	-1.41	2.17	5.80	6.32	3.22	<0.0001
**Levine**	-5.78	-5.78	4.26	4.01	4.36	2.55	<0.0001

Abbreviations: ChronoAge, chronological age; DNAmAge, DNA methylation-based epigenetic age; AgeAcce, age acceleration; SD, Standard Deviation; Max, maximum; Min, minimum; Adj. p-value, adjusted p-value.

Table S4. Data from the comparison between ChronoAge and DNAmAge and the calculation AgeAccel, estimated by each epigenetic predictor, of the subjects included in the longitudinal cohort.

**BASELINE (0 days)**											
**ChronoAge**					**DNAmAge**						
**Mean**	**Median**	**SD**	**Max**	**Min**		**Mean**	**Median**	**SD**	**Max**	**Min**	**Adj. p-value**
**48.80**	49.00	9.20	64.00	38.00	**Horvath**	56.58	56.85	8.74	75.4	44.2	0.0005
					**Hannum**	56.36	56.67	8.75	74.51	45.84	0.0006
					**Levine**	55.38	54.34	9.39	73.67	42.74	0.0006
**NUTRITIONAL KETOSIS (30 days)**											
**ChronoAge**					**DNAmAge**						
**Mean**	**Median**	**SD**	**Max**	**Min**		**Mean**	**Median**	**SD**	**Max**	**Min**	**Adj. p-value**
**48.80**	49.00	9.20	64.00	38.00	**Horvath**	44.51	44.99	8.61	61.37	31.59	0.0009
					**Hannum**	43.00	43.01	8.24	61.62	30.92	0.0043
					**Levine**	40.50	42.80	6.67	47.28	28.67	0.0005
**ENDPOINT (180 days)**											
**ChronoAge**					**DNAmAge**						
**Mean**	**Median**	**SD**	**Max**	**Min**		**Mean**	**Median**	**SD**	**Max**	**Min**	**Adj. p-value**
**48.80**	49.00	9.20	64.00	38.00	**Horvath**	45.55	45.89	7.80	61.18	36.24	0.0036
					**Hannum**	41.94	41.69	7.29	57.54	33.18	0.0036
					**Levine**	40.98	40.24	8.50	59.32	29.68	0.0022

**AgeAccel**											
	**BL**			**NK**			**EP**			**BL-NK**	**BL-EP**
	**Mean**	**Median**	**SD**	**Mean**	**Median**	**SD**	**Mean**	**Median**	**SD**	**Adj. p-value**	**Adj. p-value**
**Horvath**	7.28	5.73	4.05	-3.29	-2.77	4.00	-3.15	-2.58	3.61	<0.0001	<0.0001
**Hannum**	7.06	6.83	3.63	-6.30	-5.13	5.25	-7.36	-5.64	5.59	<0.0001	<0.0001
**Levine**	6.08	5.43	3.93	-8.79	-8.13	4.53	-8.22	-5.99	5.33	<0.0001	<0.0001

Abbreviations: ChronoAge, chronological age; DNAmAge, DNA methylation-based epigenetic age; AgeAcce, age acceleration; BL, baseline; NK, nutritional ketosis; EP, endpoint; SD, Standard Deviation; Max, maximum; Min, minimum; Adj. p-value, adjusted p-value.
